# Probing behaviors and their plasticity for the aphid *Sitobion avenae* on three alternative host plants

**DOI:** 10.1371/journal.pone.0203219

**Published:** 2018-09-05

**Authors:** Xianliang Huang, Deguang Liu, Xiaoning Cui, Xiaoqin Shi

**Affiliations:** 1 State Key Laboratory of Crop Stress Biology for Arid Areas (Northwest A&F University), Yangling, Shaanxi, China; 2 College of Plant Protection, Northwest A&F University, Yangling, Shaanxi, China; University of Saskatchewan College of Agriculture and Bioresources, CANADA

## Abstract

Insects may develop different behavioral phenotypes in response to heterogeneous environments (e.g., host plants), but the plasticity of their feeding behaviors has been rarely explored. In order to address the issue, clones of the English grain aphid, *Sitobion avenae* (Fabricius), were collected from wheat, and their probing behaviors were recorded on three plants. Our results demonstrated that *S*. *avenae* individuals on the alternative plants (i.e., barley and oat) tended to have higher frequency of non-probing (Np), increased duration of the pathway phase, increased phloem salivation, and decreased phloem ingestion (E2), compared to those on the source plant (i.e., wheat), showing the resistance of barley and oat to this aphid’s feeding. This aphid showed apparently high extents of plasticity for all test probing behaviors on barley or oat. Positive selection for higher extents of plasticity in E2 duration was identified on barley and oat. The factor ‘clone’ alone explained 30.6% to 70.1% of the total variance for each behavioral plasticity, suggesting that the divergence of probing behavior plasticity in *S*. *avenae* had a genetic basis. This aphid’s fitness correlated positively with the plasticity of Np frequency and E2 frequency. Some behaviors and their corresponding plasticities (e.g., the frequency of xylem ingestion and its plasticity) were found to be correlated characters, probably reflecting the limits for the evolution of higher extents of behavioral plasticity in this aphid. The differential probing behaviors and their plasticity in *S*. *avenae* can have significant implications for the adaptation and management of aphids on different plants.

## Introduction

Insect behaviors such as foraging are highly responsive to various environments, which allows insects to rapidly acclimate to unfavorable conditions (e.g., resistant plants) [1]. Indeed, aphids (as major insect pests on various crops) can show modified feeding behaviors on host plants with variable nutrient and secondary metabolite contents. For example, the corn leaf aphid, *Rhopalosiphum maidis* (Fitch), showed prolonged probing behaviors, increased phloem salivation, and reduced xylem sap ingestion on barley than on miscanthus [2]. The soybean aphid (*Aphis glycines* Matsumura) initiated less probes and sieve element phases on soybean cultivars with *Rag* resistant genes than on susceptible cultivars [3–4]. Such behavioral changes were also well illustrated in studies on *Diuraphis noxia* (Mordvilko), *Acyrthosiphon kondoi* (Shinji), *Myzus persicae* (Sulzer), *Rhopalosiphum padi* (L.) and *Aphis gossypii* (Glover) [5–11]. In addition, transition schemes of waveform events for feeding aphids varied between susceptible and resistant plants [2,12]. These studies indicate that aphids can show phenotypic plasticity (i.e., the ability of an individual to alter its phenotype in response to changes in environmental conditions [13]) in various feeding behaviors in order to enhance their fitness on unfavorable host plants (i.e., alternative environments). Following Gorur et al. [13], and Kelly et al. [14], behavioral plasticity is defined as variation in behavioral expression of a genotype that occurs in response to particular (including both original and alternative) environmental conditions.

Behavioral plasticity has been well characterized in some social insects such as the harvester ant (*Pogonomyrmex occidentalis*), honeybee (*Apis mellifera*), and bumblebee (*Bombus terrestris*) [15–18]. In the fruit fly *Drosophila melanogaster* and parasitoid wasp *Hyposoter horticola*, behavioral plasticity was also identified [19–20]. Similarly, the black bean aphid (*Aphis fabae*) showed high plasticity in host choice behaviors [13]. High behavioral variability was observed for the melon aphid (*A*. *gossypii*) on melon with *Vat* gene [21], and for the black currant-lettuce aphid [*Nasonovia ribisnigri* (Mosely)] on *Nr* -carrying lettuce [22]. Therefore, behavioral plasticity might play a critical role in aphid adaptation to resistant host plants [22]. Studies in this respect have been rare, although phenotypic plasticity has been considered as a powerful means of adaption to fluctuating environments, and received increasing attention in ecological and evolutionary studies [23–26].

As a worldwide cereal pest, the English grain aphid [*Sitobion avenae* (Fabricius)] survives on many plants in the Poaceae and shows a certain degree of specialization on some cereals (i.e., wheat, barley and oat) [27–29]. A few studies have showed modified feeding behaviors (e.g., increased time of xylem sap ingestion and reduced duration of phloem sap ingestion) of *S*. *avenae* on some resistant wheat lines by using the EPG (electrical penetration graph) technique (EPG waveforms can reflect different feeding behaviors of herbivorous insects with piercing mouthparts) [30–33]. Hence, this aphid provides a good model to examine behavioral plasticity and its relationships with fitness and adaptation of aphids on different plants. To our knowledge, this is the first extensive study on behavioral trait plasticity of the important cereal aphid (*S*. *avenae*). In the present study, we tested *S*. *avenae* clones on wheat, barley and oat, and recorded their probing behaviors using the EPG (electrical penetration graph) technique. Specifically, the aims of this study were to: 1) identify differences in *S*. *avenae*’s probing behaviors and their plasticity using three cereal plants (i.e., wheat, barley and oat); 2) evaluate if probing behaviors (e.g., pathway phase and phloem salivation) and their plasticity are correlated characters; and 3) explore the relationships between probing behavioral plasticity and the fitness (i.e., lifetime fecundity) of *S*. *avenae*.

## Materials and methods

### Aphid clones and plants

Aphid clones of *S*. *avenae* were sampled from wheat fields near the campus of Northwest A&F University (Yangling, Shaanxi Province, China) in May 2014. No particular permits were required for aphid collection at the above-mentioned sites, and our target insect (i.e., *S*. *avenae*) is not endangered or protected. Collected *S*. *avenae* clones were enclosed separately on wheat (*Triticum aestivum* L. cv. Aikang 58) seedlings to establish lab colonies following the method of Dai et al. [24]. Wheat seedlings were planted in plastic pots (diameter: 6 cm) filled with turfy soil, vermiculite and perlite (4:3:1, v / v / v). Aphid clones were genotyped by using six microsatellite loci (S 4∑, S 5.L, Sm10, Sm12, Sm17, Sm17b) following the method of Huang et al. [34]. Through this approach, seven genetically distinct clones of *S*. *avenae* were identified and used in this study. In order to conduct reciprocal experiments (under both original and alternative environments) and analyze plastic changes in behavior after they were transferred to alternative environments (i.e., barley and oat), test *S*. *avenae* clones were reared on the wheat cultivar Aikang 58 (i.e., the original environment) in common laboratory conditions for at least three generations before the initiation of the experiment. This is also a common practice to minimize or eliminate confounding effects from different original environments (e.g., different wheat cultivars and variable microclimates) [29].

### Life history bioassays

The life-history test with seven *S*. *avenae* clones was conducted as described previously in [35]. Briefly, when reaching one- or two-leaf stage, single seedlings of wheat (*T*. *aestivum* cv. Aikang 58), barley (*Hordeum vulgare* L. cv. Xian 91–2), and oat *(Avena sativa* L. cv. Sandle) received one new-borne, age-synchronized first instar nymph that was transferred from wheat. In order to prevent aphid escape, each pot of seedlings was enclosed in a plastic container (diameter: height = 6: 15 cm). Test plants were placed in environmental growth chambers with temperature 22 ± 1°C, photoperiod 16: 8 (L: D), and relative humidity 65 ± 2%. The developmental instars of test aphid nymphs were determined by checking twice daily for the occurrence of molting and existence of exuviae. We monitored each aphid individual daily until its death, and molting, birth and mortality events were recorded with offspring removed at the same time. The bioassay on *S*. *avenae* clones was replicated three or four times under each treatment.

### Electrical penetration graph (EPG) assays

The EPG technique was used to monitor feeding behaviors of *S*. *avenae* on the above-mentioned cultivars of wheat, barley, and oat. The EPG assays were conducted according to the methods described in [2] using a Giga-8 DC EPG System (EPG Systems, Wageningen, Netherlands). Briefly, a copper electrode was attached to the dorsum of test aphid individuals by conductive silver glue (EPG systems, Wageningen, The Netherlands), and another electrode was inserted into the pot. The probing signals were recorded by the amplifier of the EPG system. In this study, first-instar nymphs fed separately on wheat, barley, and oat as described earlier until they reached the adult stage. After being starved for 1 h, one- to two-days old wingless adults were tested on 7-d old intact plants in a climate-controlled room with the aforementioned environmental conditions. EPG waveforms were recorded for 8 h for each aphid individual. Four to six replications of each aphid clone were performed. The EPG assays were conducted from 8:00 to 16:00 o’clock each day, and same numbers of replicates were conducted on each plant at each time slot. However, a few tests produced no data due to the aphid attachment issue (i.e., the electrode was not sufficiently attached to the test aphid so that the aphid could escape), resulting in unequal numbers of replicates on three plants. A total of 37, 37 and 28 EPG recordings for the seven aphid clones were obtained on wheat, barley and oat, respectively.

### Statistical analysis

As described in [36], the software Stylet^+^ was used to analyze the following waveforms. The waveform Np refers to the non-probing phase, C to the pathway phase, E1 to phloem salivation, E2 to passive sap ingestion from the phloem, G to active sap ingestion from the xylem, and F to stylet derailment. The frequency and duration for each waveform were calculated. The frequency of Np, the duration of G, the frequency of F and the duration of F were analyzed with Kruskal-Wallis tests, and means were separated with Dunn’s tests at α = 0.05 in SAS [37]. The other traits were analyzed with one-way analysis of variance (ANOVA), and means were then separated with Tukey tests at α = 0.05 in SAS. Sequences of waveforms were analyzed for each plant, and the probability of transition from a particular waveform to another was calculated. The proportions obtained on three test plants were compared with Chi-square tests in SAS.

The amount of plasticity for probing behaviors of *S*. *avenae* was assessed by calculating the coefficient of variation (*CV*) following the method in [24]. Data were then analyzed with two-way analysis of variance (ANOVA), and effects of ‘plant’, ‘clone’, and the interaction between ‘plant’ and ‘clone’ were estimated. Means were separated with Tukey tests at α = 0.05 following a significant ANOVA in SAS. When needed, data were log transformed to meet the requirements of normality and homoscedasticity for these analyses. The PROC CORR procedure was used to assess the association between a particular probing behavior and its corresponding plasticity by the Spearman rank-order correlation analysis and Hoeffding independence test in SAS.

In order to assess the relationship between behavioral plasticity and *S*. *avenae*’s fitness, the lifetime fecundity for aphid clones was recorded on each plant. The relative fecundity of each aphid clone was calculated through dividing the clone’s lifetime fecundity by the mean of all test lines under each treatment. The relationship between probing behavior plasticity and relative fecundity was determined using Pearson correlation analyses in SAS. The Proc REG procedure in SAS was used to determine the selection differential and gradient (i.e., selection strength of alternative plants) for each behavioral plasticity as described in [24].

## Results

### Effects of plant species on life history traits

The developmental time of first instar nymphs for *S*. *avenae* clones was longest on barley, and lowest on wheat (Table 1; *F* = 18.35; df = 2, 66; *P* < 0.001). The developmental time of second instar nymphs on oat was longer than that on wheat (*F* = 4.99; df = 2, 66; *P* < 0.01). Compared to barley or oat, clones of *S*. *avenae* on wheat showed shorter developmental time of third (*F* = 25.13; df = 2, 66; *P* < 0.001) and fourth (*F* = 10.87; df = 2, 66; *P* < 0.001) instar nymphs, and the total developmental time of nymphs was also shorter on wheat (*F* = 56.58; df = 2, 66; *P* < 0.001). Lifetime fecundity of *S*. *avenae* clones was highest on wheat, but lowest on oat (*F* = 89.26; df = 2, 66; *P* < 0.001).

**Table 1:** Developmental time and fecundity for *Sitobion avenae* clones on wheat, barley and oat.

Traits	Wheat	Barley	Oat
DT1 (d)	1.91 ± 0.04 C	2.71 ± 0.10 A	2.33 ± 0.14 B
DT2 (d)	1.94 ± 0.04 B	2.10 ± 0.07 AB	2.24 ± 0.10 A
DT3 (d)	1.50 ± 0.07 B	2.05 ± 0.05 A	2.00 ± 0.07 A
DT4 (d)	1.91 ± 0.08 B	2.62 ± 0.13 A	2.43 ± 0.15 A
DT 5(d)	7.26 ± 0.05 B	9.48 ± 0.18 A	9.00 ± 0.24 A
Lifetime fecundity	76.78 ± 1.52 A	48.52 ± 3.07 B	35.67 ± 2.38 C

Note: DT1-DT4, developmental time of 1^st^ -4^th^ instar nymphs; DT5, total developmental time of nymphs; different letters following data within a row indicate significant differences at the *P* < 0.05 level, ANOVA followed by Tukey tests.

### Effects of plant species on probing and ingestion behaviors

Clones of *S*. *avenae* showed a higher frequency of the non-probing phase (Np) on barley than on wheat (Table 2; Kruskal-Wallis test: *χ*^*2*^ = 7.21; df = 2; *P* = 0.027), while the duration of Np had no significant differences among the three test plants (i.e., wheat, barley and oat). The frequency of the pathway phase (C) for *S*. *avenae* clones was also higher on barley than on wheat (*F* = 4.45; df = 2, 94; *P* = 0.014). Compared to that on wheat, a longer duration of C on barley or oat was found (*F* = 12.68; df = 2, 94; *P* < 0.001). The occurrence of stylet derailments (F) for *S*. *avenae* clones was the most frequent on barley (Kruskal-Wallis test: *χ*^*2*^ = 16.63; df = 2; *P* < 0.001), and its duration was the longest on barley (Kruskal-Wallis test: *χ*^*2*^ = 18.02; df = 2; *P* = 0.001). The frequency of phloem salivation (E1) showed no significant differences among plants, but the duration of E1 was longer on oat than on wheat (*F* = 5.57; df = 2, 94; *P* < 0.01). Among the three plants, *S*. *avenae* clones on wheat presented highest frequency (*F* = 5.46; df = 2, 94; *P* < 0.01) and longest duration (*F* = 19.48; df = 2, 94; *P* < 0.001) of E2 (phloem sap ingestion). The duration of the xylem ingestion phase (G) was longer on barley than on wheat (Kruskal-Wallis test: *χ*^*2*^ = 7.29; df = 2; *P* = 0.026).

**Table 2:** Frequency and duration^#^ (mean ± SE) of probing behaviors for *Sitobion avenae* clones on wheat, barley and oat.

EPG parameters	Wheat ^§^	Barley	Oat
Np frequency	9.00 ± 0.81 b	12.76 ± 0.91 a	11.43 ± 1.92 ab
Np duration	53.99 ± 8.67 A	56.04 ± 8.50 A	63.11 ± 12.05 A
C frequency	11.30 ± 0.90 B	16.22 ± 1.08 A	14.83 ± 2.13 AB
C duration	107.04 ± 7.79 B	179.36 ± 11.38 A	176.75 ± 18.78 A
F frequency	0.16 ± 0.06 b	0.84 ± 0.18 a	0.13 ± 0.07 b
F duration	5.67 ± 2.44 b	45.79 ± 9.63 a	5.63 ± 3.35 b
E1 frequency	3.97 ± 0.45 A	3.65 ± 0.43 A	4.48 ± 0.62 A
E1 Duration	30.59 ± 4.21 B	42.96 ± 6.93 AB	62.32 ± 8.08 A
E2 frequency	1.97 ± 0.21 A	1.24 ± 0.11 B	1.35 ± 0.21 B
E2 duration	274.07 ± 14.15 A	131.47 ± 17.02 B	156.17 ± 26.18 B
G frequency	0.05 ± 0.04 A	0.32 ± 0.10 A	0.30 ± 0.19 A
G duration	0.34 ± 0.28 b	13.33 ± 4.49 a	5.94 ± 3.62 ab

^#^, durations expressed in minutes

^**§**^, a total of 37, 37 and 28 EPG recordings were obtained on wheat, barley and oat, respectively

data in a row followed by different letters indicate significant differences at α = 0.05; upper case letters indicate ANOVA followed by Tukey tests; lower case letters indicate Kruskal–Wallis tests followed by Dunn’s tests; 05, Np, non-probing; C, pathway phase; E1, phloem salivation; E2, phloem sap ingestion; G, xylem sap ingestion; F, stylet derailment.

**Fig 1:**
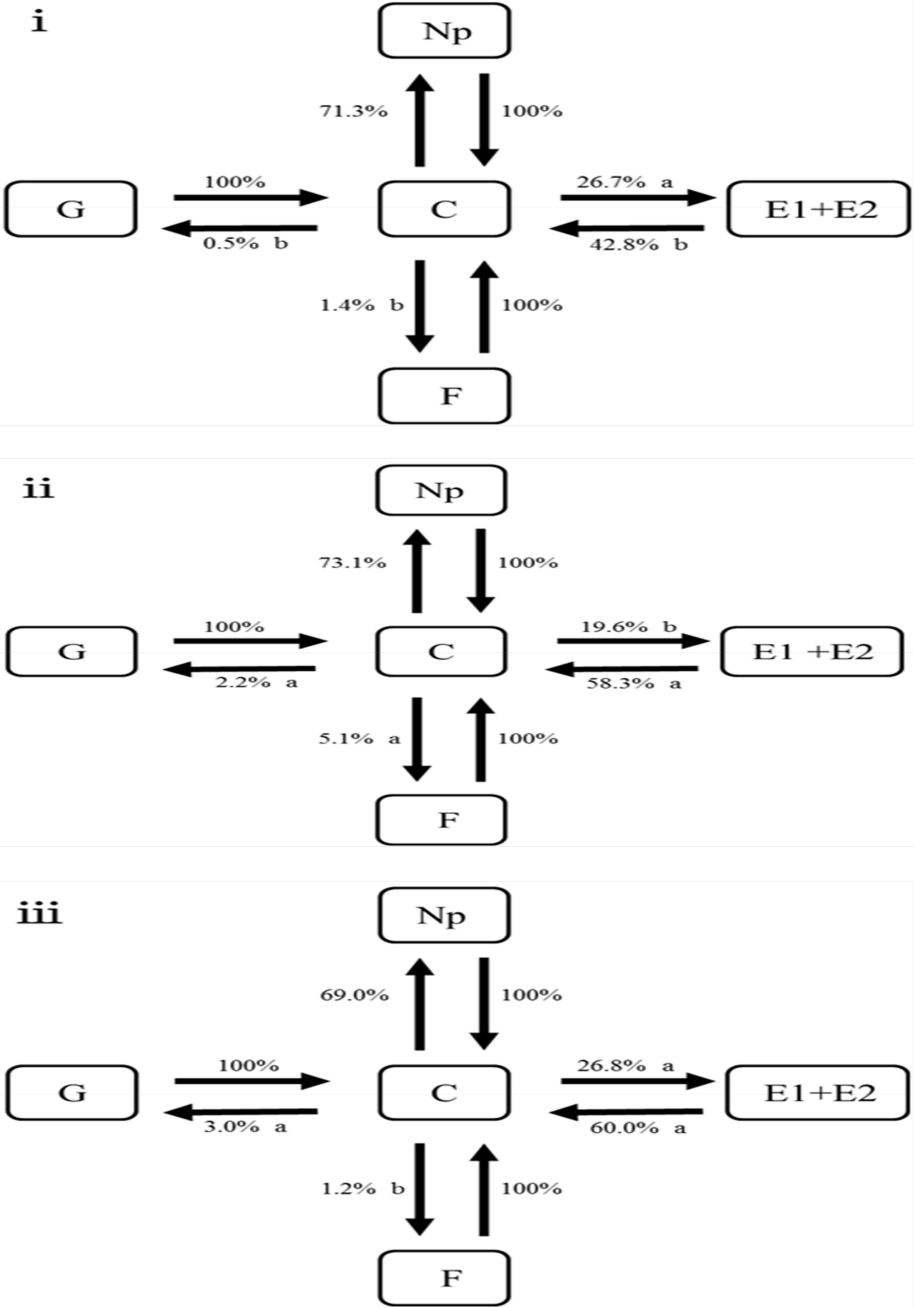
Comparisons of probing behavioral patterns for *Sitobion avenae* clones on wheat (i), barley (ii), and oat (iii). (Values next to the arrows represent the probability of individuals entering a certain feeding step; probabilities followed by different lower case letters indicate significant differences between plants identified with Chi-square tests at *P* < 0.05; Np, non-probing; C, pathway phase; E1, phloem salivation; E2, phloem ingestion; G, xylem ingestion; F, stylet derailment).

The sequences of probing behaviors for *S*. *avenae* clones varied among the three test plants (Fig 1). After the pathway phase, aphid individuals showed a lower propensity to enter the phloem phase (E1 and E2) on barley (19.6%) than on wheat (26.7%; *χ*^*2*^ = 7.15, df = 1, *P* < 0.01) or oat (26.8%; *χ*^*2*^ = 6.41, df = 1, *P* < 0.05). Aphid individuals had a higher propensity to undertake a transition from the pathway phase to stylet derailments on barley (5.1%) than on wheat (1.4%; *χ*^*2*^ = 9.28, df = 1, *P* < 0.01) or oat (1.2%; *χ*^*2*^ = 8.95, df = 1, *P* < 0.01). Aphid individuals feeding on wheat showed a lower propensity (0.5%) to start xylem ingestion after the pathway phase compared to those feeding on barley (2.2%; *χ*^*2*^ = 4.88, df = 1, *P* < 0.05) or oat (3.0%; *χ*^*2*^ = 7.47, df = 1, *P* < 0.01). The probability of switching from the phloem phase back to the pathway phase was also lower on wheat (42.8%) than on barley (58.3%; *χ*^*2*^ = 8.03, df = 1, *P* < 0.01) or oat (60.0%; *χ*^*2*^ = 8.67, df = 1, *P* < 0.01). However, similar probabilities of aphid individuals returning to the non-probing phase after the pathway phase were found on all three plants. The same pattern was also found for transitions from Np to C, from F to C, and from G to C.

### Plasticity of probing and ingestion behaviors and selective effect of alternative plants

**Fig 2:**
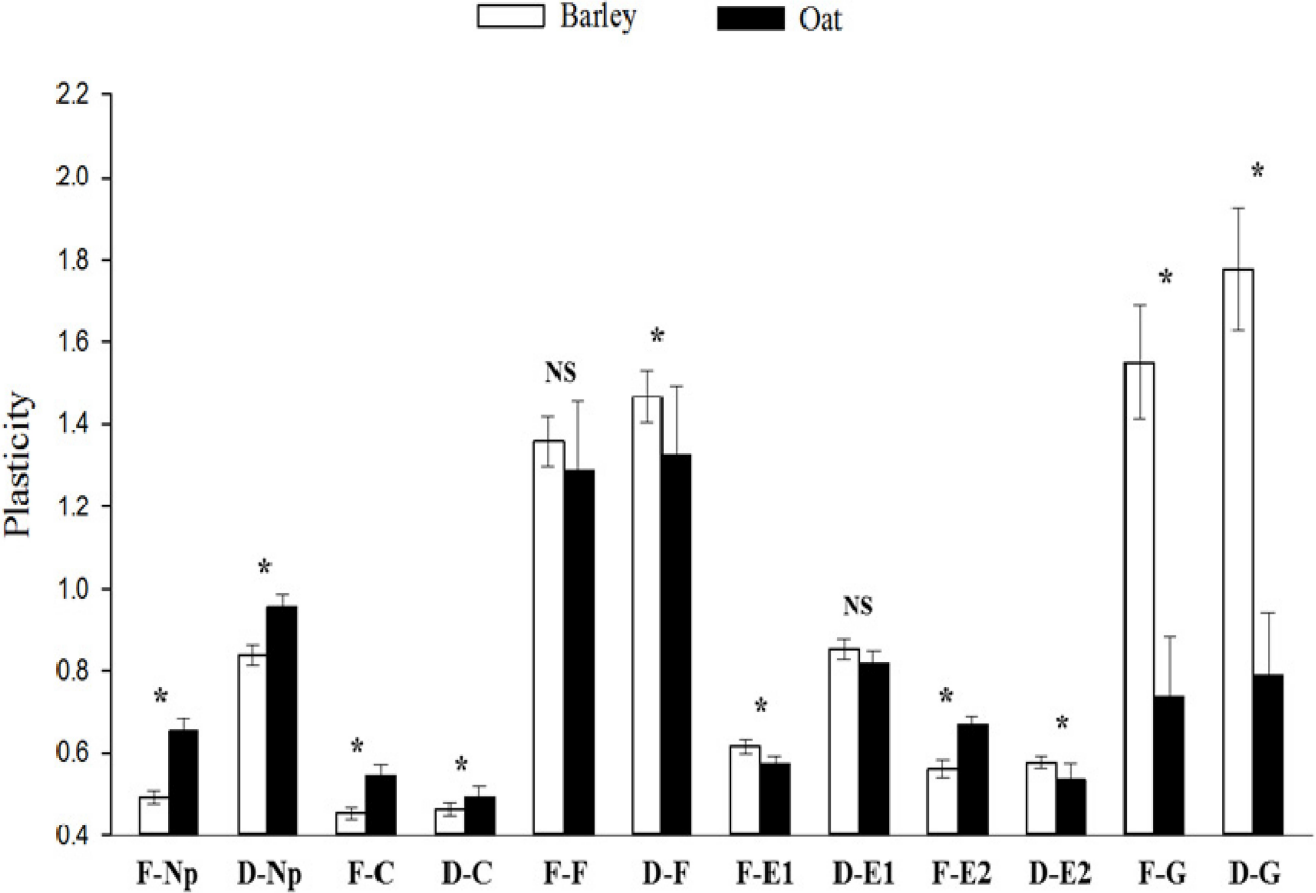
Comparisons of probing behavioral plasticities (mean ± SE) for *Sitobion avenae* clones on barley and oat. (F, frequency; D, duration; Np, non-probing; C, pathway phase; E1, phloem salivation; E2, phloem sap ingestion; G, xylem sap ingestion; F, stylet derailment; * and NS, significant and non-significant differences between plants identified with two-way ANOVA at *P* < 0.05).

Compared to those on barley, *S*. *avenae* clones on oat showed a higher plasticity level in frequency of Np (Fig 2; *F* = 138.81; df = 1, 132; *P* < 0.001), duration of Np (*F* = 32.72; df = 1, 132; *P* < 0.001), frequency of C (*F* = 43.66; df = 1, 132; *P* < 0.001), duration of C (*F* = 6.79; df = 1, 132; *P* = 0.010), and frequency of E2 (*F* = 43.41; df = 1, 132; *P* < 0.001). However, in comparison with barley, a lower plasticity level on oat was found in frequency of E1 (*F* = 7.80; df = 1, 132; *P* = 0.006), duration of E2 (*F* = 5.73; df = 1, 132; *P* = 0.018), frequency of G (*F* = 112.12; df = 1, 132; *P* < 0.001), duration of G (*F* = 174.36; df = 1, 132; *P* < 0.001), and duration of F (*F* = 11.30; df = 1, 132; *P* = 0.001). No significant differences between barley and oat were found in the plasticity of F frequency and E1duration.

Variance components (i.e., ‘plant’, ‘clone’, and ‘plant × clone’) for plasticity of *S*. *avenae*’s probing behaviors were estimated (Table 3). ‘Plant’ accounted for a significant proportion of the total variance for plasticities of all probing and ingestion behaviorss but those of F frequency and E1 duration, showing significant impacts of alternative plants on behavioral plasticity. ‘Clone’ and the interaction between ‘plant’ and ‘clone’ showed significant effects for every behavioral character. ‘Clone’ alone explained 30.56% - 70.09% of the total variance for plasticity of each probing behavior. The interaction of ‘clone’ and ‘plant’ contributed 8.12% - 50.13% to the total variance for plasticity of test behavioral traits.

**Table 3:** Estimates of variance components for probing behavioral plasticities of *Sitobion avenae* clones showing main effects of plant, clone and interactions.

EPG parameters	Variance component															
	Plant				Clone				Plant×Clone				Error			
	df	*F*	*P*	% total	df	*F*	*P*	% total	df	*F*	*P*	% total	df	*F*	*P*	% total
Np frequency	1	138.81	**< 0.001**	**15.86**	6	91.01	**< 0.001**	**62.42**	6	11.84	**< 0.001**	**8.12**	120	-	**-**	13.60
Np duration	1	32.72	**< 0.001**	**6.63**	6	40.84	**< 0.001**	**49.69**	6	16.07	**< 0.001**	**19.55**	120	-	-	24.13
C frequency	1	43.66	**< 0.001**	**7.12**	6	65.50	**< 0.001**	**64.11**	6	9.55	**< 0.001**	**9.34**	120	-	-	19.41
C duration	1	6.79	**0.010**	**0.67**	6	116.40	**< 0.001**	**68.97**	6	31.39	**< 0.001**	**18.60**	120	-	-	11.75
F frequency	1	2.69	0.104	0.14	6	214.52	**< 0.001**	**67.24**	6	84.25	**< 0.001**	**26.41**	120	-	-	6.22
F duration	1	11.30	**0.001**	**0.55**	6	238.21	**< 0.001**	**69.45**	6	83.09	**< 0.001**	**24.22**	120	-	-	5.78
E1 frequency	1	7.80	**0.006**	**2.22**	6	25.61	**< 0.001**	**43.83**	6	11.68	**< 0.001**	**19.99**	120	-	-	33.95
E1 duration	1	3.32	0.071	0.57	6	68.62	**< 0.001**	**70.09**	6	8.90	**< 0.001**	**9.09**	120	-	-	20.26
E2 frequency	1	43.41	**< 0.001**	**8.38**	6	38.52	**< 0.001**	**44.61**	6	20.75	**< 0.001**	**24.04**	120	-	-	22.97
E2 duration	1	5.73	**0.018**	**0.89**	6	32.90	**< 0.001**	**30.56**	6	53.97	**< 0.001**	**50.13**	120	-	-	18.42
G frequency	1	112.12	**< 0.001**	**11.02**	6	81.13	**< 0.001**	**47.83**	6	49.98	**< 0.001**	**29.46**	120	-	-	11.69
G duration	1	174.36	**< 0.001**	**14.08**	6	111.55	**< 0.001**	**54.04**	6	45.99	**< 0.001**	**22.28**	120	-	-	9.61

Notes: Np, non-probing; C, pathway phase; E1, phloem salivation; E2, phloem sap ingestion; G, xylem sap ingestion; F, stylet derailment

Selective effects of alternative plants on probing behavioral plasticity for *S*. *avenae* clones were analyzed (Table 4). For aphids feeding on barley, the selection differential for plasticity in frequency and duration of F was significantly negative, but it was significantly positive for duration of E2. The only significant selection gradient for *S*. *avenae* clones feeding on barley was found for plasticity of F duration. Oat showed significantly selective effects on plasticity of E2 duration only, for which the selection differential and gradient were both positive.

**Table 4:** Selection differentials and gradients for probing behavioral plasticities of *Sitobion avenae* clones on two alternative plants (i.e., barley and oat).

Parameters	Barley		Oat	
	Differential	Gradient	Differential	Gradient
Np frequency	-0.1753	0.1060	0.1962	0.2110
Np duration	-0.0441	0.0842	-0.0281	0.0234
C frequency	-0.1876	-0.2319	0.1341	-0.0737
C duration	-0.1864	-0.1691	-0.0509	-0.2381
F frequency	**-0.3920** ***	-0.0098	-0.0294	-0.0123
F duration	**-0.4939** ***	**-0.4107** *	-0.0535	-0.1300
E1 frequency	0.0604	0.1676	0.0065	0.1065
E1 Duration	0.2203	0.0182	-0.2212	-0.0982
E2 frequency	-0.0470	-0.2118	0.1621	-0.1763
E2 duration	**0.2918** *	0.1945	**0.4799** ***	**0.6148** ***
G frequency	-0.0980	0.3388	-0.0162	0.1065
G duration	-0.2123	-0.3634	-0.1690	-0.0631

Np, non-probing; C, pathway phase; E1, phloem salivation; E2, phloem ingestion; G, xylem ingestion; F, stylet derailment

*, *P* < 0.05

***, *P* < 0.001

significant effects highlighted in bold type.

### Associations between probing and ingestion behaviors and their plasticity

**Fig 3:**
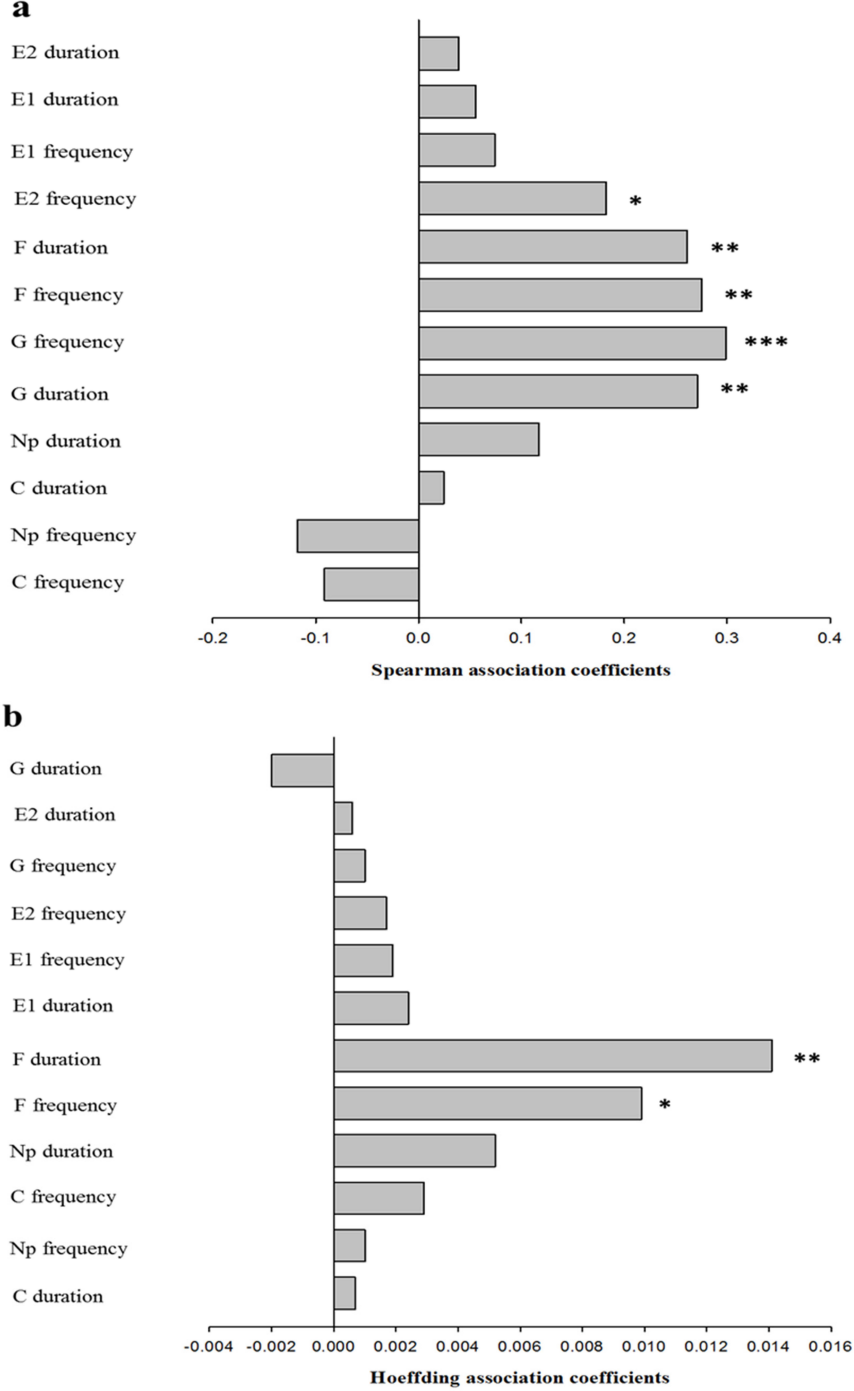
**Spearman (a) and Hoeffding (b) association coefficients between probing behaviors and their plasticities for *Sitobion avenae* clones** (Np, non-probing; C, pathway phase; E1, phloem salivation; E2, phloem ingestion; G, xylem ingestion; F, stylet derailment. *, *P* < 0.05; **, *P* < 0.01; ***, *P* < 0.001).

Using both Spearman correlation analysis and Hoeffding independence tests, both F frequency (Fig 3A: Spearman correlation, ρ = 0.2753, *P* < 0.01; Fig 3B: Hoeffding test, *D* = 0.0099, *P* < 0.05) and F duration (Spearman correlation: ρ = 0.2611, *P* < 0.01; Hoeffding test: *D* = 0.0141, *P* < 0.01) for *S*. *avenae* clones were found to be significantly correlated with their corresponding plasticity. The dependence of G frequency (ρ = 0.2986, *P* < 0.001), G duration (ρ = 0.2712, *P* < 0.01) and E2 frequency (ρ = 0.1825, *P* < 0.05) on their corresponding plasticity was also found based on Spearman correlation analyses, although such associations were non-significant based on Hoeffding independence tests. All the other test behavioral characters (e.g., E1 frequency, E2 duration, and C duration) were found to be independent of their corresponding plasticity. Such results suggest that the independence of particular behavioral plasticity from the behavior involved was character specific.

### Relationships between behavioral plasticity and relative fitness

**Fig 4:**
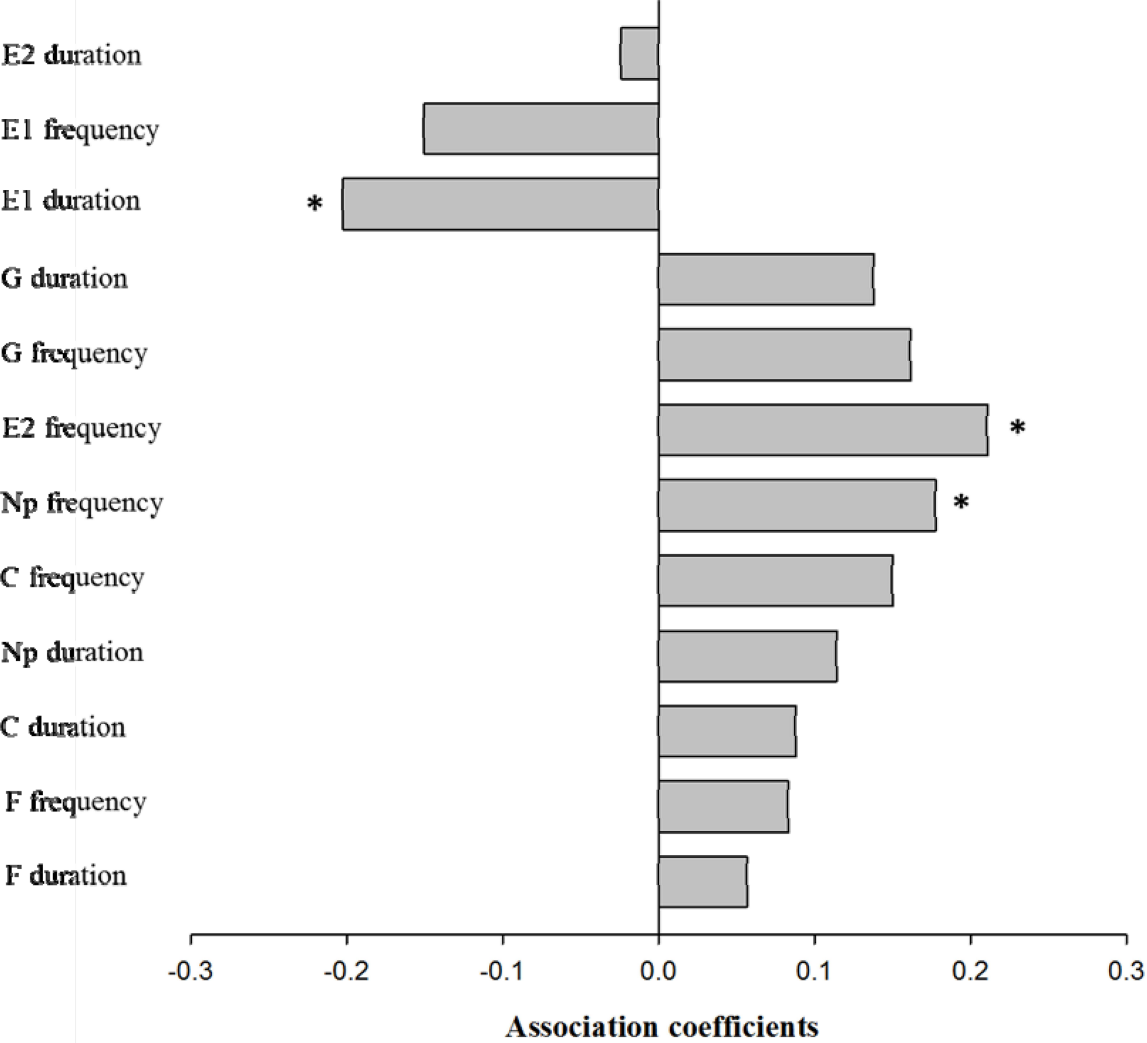
Pearson correlations between relative fitness and probing behavioral plasticities for *Sitobion avenae* clones. (Np, non—probing; C, pathway phase; E1, phloem salivation; E2, phloem ingestion; G, xylem ingestion; F, stylet derailment. *, *P* < 0.05).

The relative fitness of *S*. *avenae* clones was found to be significantly and positively correlated with the plasticity of E2 frequency (Fig 4; *r* = 0.2111, *P* < 0.05) and Np frequency (*r* = 0.1778, *P* < 0.05). The correlation between relative fitness and the plasticity of E1 duration was significantly negative (r = -0.2024, *P* < 0.05). The relationship between the plasticity of all other test behavioral characters and *S*. *avenae*’s relative fitness was non-significant.

## Discussion

### Probing and ingestion behaviors on three plants

When transferred from the source plant (i.e., wheat) onto the alternative plants (i.e., barley and oat), *S*. *avenae* clones tended to have higher frequency and longer duration of the pathway phase, indicating transient difficulties from the sieve element puncture activity to phloem salivation (E1) on barley and oat. This is consistent with the findings that the Russian wheat aphid (*D*. *noxia*) shows higher frequency of probes and longer salivation duration on resistant plants (e.g., oat or resistant barley cultivar ‘STARS-9301B’) than on susceptible plants (e.g., susceptible barley cultivar ‘Morex’) [38–39]. Clones of *S*. *avenae* also tended to have prolonged phloem salivation and reduced phloem sap ingestion (E2) on the alternative plants, indicating the initial difficulties for this aphid to initiate phloem sap ingestion and a reduced ability to suppress the phloem wound responses (e.g., protein clogging inside a sieve element) of both plants [40]. Clones of *S*. *avenae* showed a tendency to have an increased duration of xylem sap ingestion after being switched onto barley or oat, and this could be due toe a higher intensity of osmotic stress resulting from the high C:N ratio diet of both plants [2,41–42]. This aphid’s reduced phloem feeding and increased xylem feeding on certain plants could also be attributed to physical barriers (e.g., sclerenchymatous rings) or secondary metabolites (e.g., flavonoid glycosides) [43–45]. Compared to wheat or oat, both longer durations and higher frequencies of stylet derailments were found for *S*. *avenae* clones feeding on barley, showing mechanical difficulties in the stylet pathway in the mesophyll of this plant [33]. These mechanical difficulties might be derived from differences in the thickness of bundle sheaths, and the arrangement of tonoplast, and cell wall composition in the plants involved [2,46–47]. Overall, *S*. *avenae* clones tended to have lower frequency of non-probing (Np), reduced pathway phase, decreased phloem salivation, and increased phloem sap ingestion on wheat (i.e., the source plant), showing that wheat was most susceptible to this aphid among the three test plants.

Additionally, the sequences of probing behaviors for *S*. *avenae* varied among three plants. For example, *S*. *avenae* individuals feeding on the alternative plants (i.e., barley and oat) had a higher propensity to undertake transitions from the pathway phase (C) to xylem ingestion and from C to stylet derailments, compared to those feeding on the source plant (i.e., wheat). A higher probability of transition from the phloem phase (phloem salivation and sap ingestion) to C was also found for *S*. *avenae* feeding on the alternative plants than on the source plant. The pattern of probing behavioral sequences on the three plants provides additional evidence that the alternative plants were more resistant to the feeding of *S*. *avenae* than the source plant. The among-plant differences in probing behaviors (or behavioral sequences) of *S*. *avenae* may be due to the features of the leaf surface (e.g., foliar trichome density and epicuticular wax) or phloem-located cues (e.g., secondary metabolites and phagodeterrents) [2,4,48–51]. Further studies are required to clarify the resistant factors in barley or oat, which might be useful in cultural control of *S*. *avenae* [28]. Thus, *S*. *avenae* can respond to the differential resistant factors in host plants by developing alternative behavioral phenotypes. This is not unexpected because some *S*. *avenae* clones have shown certain degree of specialization on wheat, barley or oat in our previous studies [28–29]. It will be interesting to explore the impact of different behavioral phenotypes on adaptation (or specialization) of *S*. *avenae* clones on alternative plants in the future.

### Patterns of behavioral plasticity and its relationship with fitness in *S*. *avenae*

In this study, *S*. *avenae* clones feeding on the two alternative plants (i.e., alternative environments) showed apparently high extents of plasticity for all test probing behaviors, especially for stylet derailments and xylem sap ingestion on barley, and for stylet derailments on oat. This aphid presented relatively higher plasticities in phloem salivation, xylem sap ingestion and stylet derailment on barley than on oat, whereas it showed significantly higher plasticities for feeding activities of non-probing (Np) and the pathway phase (C) on oat than on barley. This suggests a host plant-specific pattern of probing behavioral plasticity for *S*. *avenae*. So far, relatively little is known about how evolutionary processes shape the intraspecific variation in behavioral phenotypes, and if phenotypic plasticity of behaviors for different organisms has a genetic basis [1].

Our study showed that ‘clone’ (i.e., different *S*. *avenae* genotypes from wheat) explained a significant proportion (30.6% - 70.1%) of the total variance for plasticity of each behavioral trait, meaning that different genotypes of *S*. *avenae* can have differential extents of behavioral plasticity. This suggests that the divergence of behavioral plasticity in *S*. *avenae* could have a genetic basis. We also found that phenotypic plasticity of vital life-history traits (e.g., developmental time and fecundity) for *S*. *avenae* had a clear genetic basis in our previous study [24]. Even though genetic models (i.e., pleiotropy, epistasis, and overdominance) have been used to explain phenotypic plasticity, the genetic mechanisms of plastic responses are poorly understood [52–53]. Aphid populations often present a high degree of heterozygote excess in the field [34,54–55]. Thus, the overdominance model, which predicts that plasticity increases with decreasing heterozygosity, may be able to explain the pattern of plasticity for probing behaviors of *S*. *avenae*. However, all our test aphid clones came from a single plant (i.e., wheat), instead of all the three test plants. This might not represent the actual status for the extents of heterozygote excess in *S*. *avenae* in nature. Thus, use of wheat clones only might result in a bias in test results, presenting a limitation of this study. A recent study has shown that the differential regulation of genetic pathways (e.g., those involving the gene *foraging*) is likely a key mechanism involved in plasticity of insect behaviors [15]. Another molecular mechanism of behavioral plasticity in insects involves biochemical changes in the levels of neuromodulators (e.g., neuropeptides) regulated by the neuropeptide NPF/nitric oxide (NO) pathway [56]. Future studies using more *S*. *avenae* genotyopes and more replicates for each genotype are needed to confirm the above-mentioned results, and determine the exact molecular mechanism underlying behavioral plasticity identified in this study.

Do those genotypes of *S*. *avenae* with high extents of behavioral plasticity have enhanced fitness? In the current study, we did find that the fitness of *S*. *avenae* correlated positively with the plasticity for frequencies of non-probing and phloem sap ingestion, suggesting that higher plasticity in the frequency of both behaviors can be adaptive for this aphid. However, a negative correlation between *S*. *avenae*’s fitness and the plasticity for duration of phloem salivation (E1) was identified. This means that lower plasticity of E1 duration could enhance the fitness of this aphid on alternative plants. Nonetheless, the correlations between fitness of *S*. *avenae* and behavioral plasticity tended to be positive for all probing parameters except E1 duration, E1 frequency, and duration of phloem sap ingestion (E2). Therefore, high extents of plasticity in probing behaviors can potentially enhance the fitness of this aphid. Indeed, positive selection for plasticity of E2 duration was identified on both barley and oat. This suggests that natural selection of alternative plants in the field has the potential to increase the extent of plasticity for E2 duration of *S*. *avenae* in order to maximize its fitness. However, positive selection of plasticity for other probing behaviors was not identified. This probably reflects the costs and limits of the evolution of higher extents of plasticity for probing behaviors in *S*. *avenae*. In this study, the frequency of xylem sap ingestion (G) and its plasticity, as well as the duration of G and its plasticity, were found to be correlated characters, using both Spearman correlation analyses and Hoeffding independence tests. Similarly, the plasticity for duration of stylet derailment (F), F frequency, or frequency of phloem sap ingestion was also likely a correlated character for the corresponding behavior. Therefore, the evolution of higher extents of plasticity for probing behaviors of *S*. *avenae* can be constrained by selection on their correlated characters.

In summary, this study represents the first to extensively explore probing behavior plasticity of the economically important cereal aphid, *S*. *avenae*. Studies on plasticity of aphid behaviors have been rare, and there is little evidence in the literature that phenotypic plasticity is adaptive. Our results showed that this aphid had apparently high extents of plasticity for all test probing behaviors on barley or oat. Correlations between fitness (i.e., lifetime fecundity) of *S*. *avenae* and behavioral plasticity tended to be positive for a majority of the probing and ingestion behaviors, indicating that higher plasticity in these behaviors could be adaptive for this aphid. We also found that probing behavioral plasticity of *S*. *avenae* could be genetically based. Thus, our results have ecological and evolutionary implications for this aphid in the field. In addition, probing behaviors and their plasticity in *S*. *avenae* can also have significant implications for transmission of BYDV (barley yellow dwarf virus) in the field. Future studies are needed to integrate data on behavioral and life-history trait plasticities, as well as data on behavioral and life-history traits. This will help us understand the mechanisms, costs and limits of phenotypic plasticity, and its implications for management of aphids and BYDV on different host plants.
